# Supplementary material for: Methane- and dissolved organic carbon-fueled microbial loop supports a tropical subterranean estuary ecosystem
Source: Nat Commun. 2017 Nov 28;8:1835. doi: 10.1038/s41467-017-01776-x (PMC5703975; doi:10.1038/s41467-017-01776-x)
Supplement: Supplementary file 1 — Supplementary Information [file 41467_2017_1776_MOESM1_ESM.pdf]

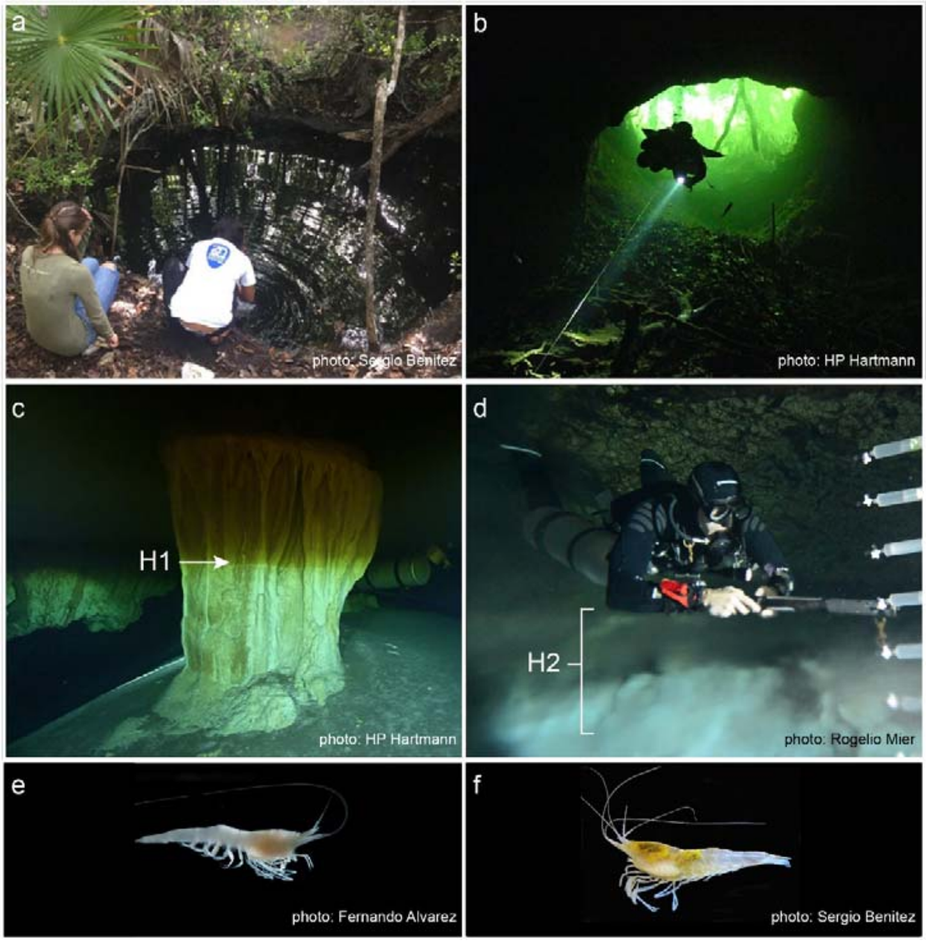

Supplementary Figure 1: **(a)** Entrance pool of Cenote Bang (20° 12.616' N 87° 30.064' W). **(b)** Subsurface vantage of Cenote Bang entrance pool, the primary locations where particulate organic detritus enters the system without being filtered by the karst. **(c)** Shallow halocline (H1) ~ 4 m depth below the groundwater table is visibly distinguished by darker coloration of the shallower water mass. **(d)** Deeper halocline (H2) located at ~ 20 m depth below the groundwater table. **(e)** *Typhlatya mitchelli* and **(f)** *Typhlatya pearsei*, the two species of stygobitic atyid shrimp present at the primary research site.

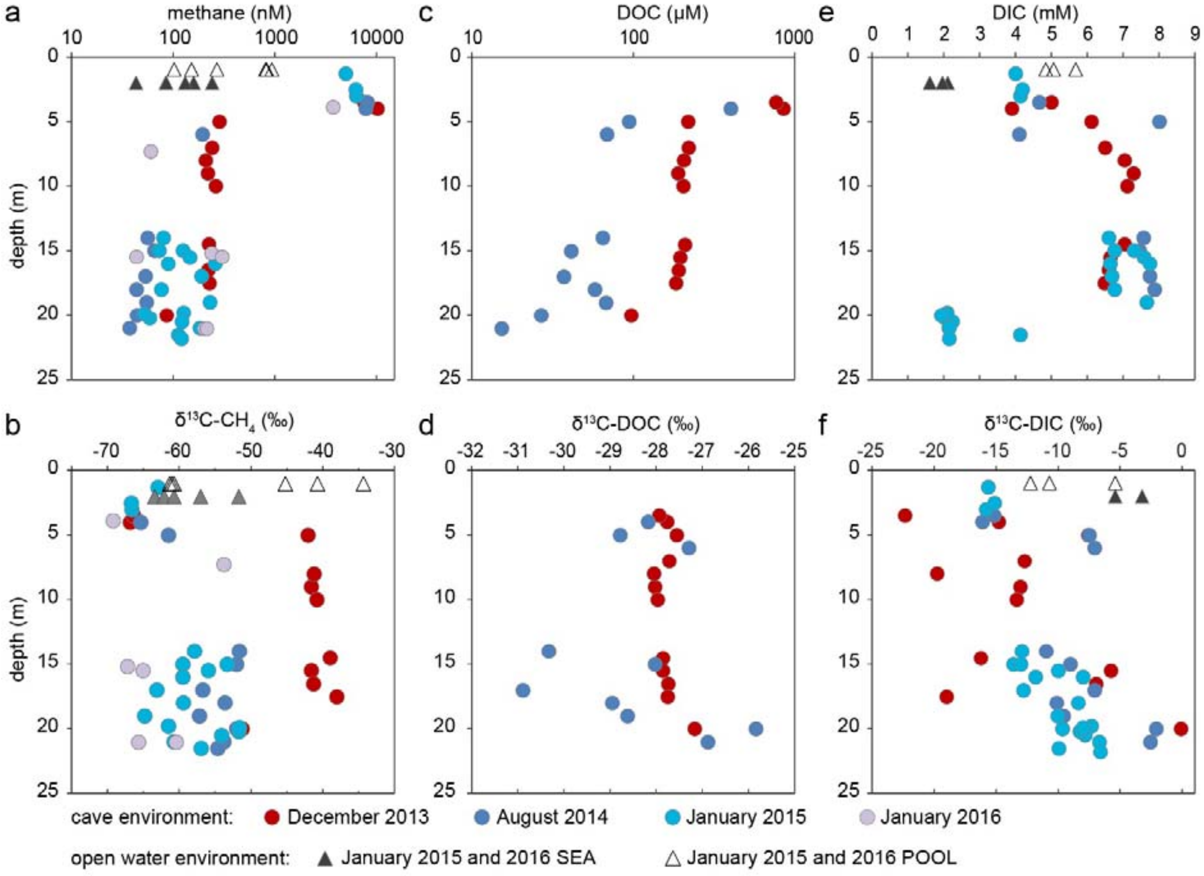

Supplementary Figure 2: Depth-property plots (concentration and carbon isotopic composition) for dissolved organic and inorganic carbon compounds. Symbols of individual data points contain the uncertainty (std. dev.) of the measured values.

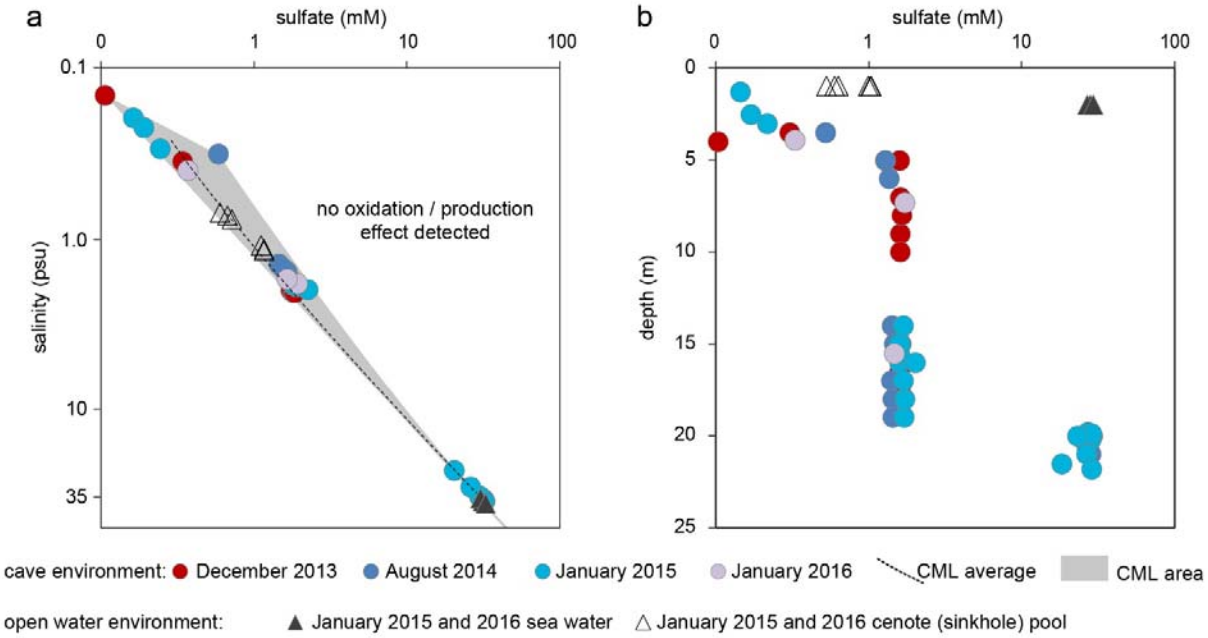

Supplementary Figure 3: Sulfate concentration at the study site. **(a)** Salinity versus sulfate. The conservative mixing calculations show no evidence of sulfate oxidation or production within the cave system. **(b)** Depth profile of sulfate concentrations. The average and total area of conservative mixing lines (CMLs; see Methods for calculations) represent the trend predicted by the mixing model if there was only physical mixing between the meteoric freshwater and saline groundwater endmembers. Production yields an excess of the constituent relative to the CML average and area, while consumption results in depletion. Symbols of individual data points contain the uncertainty (std. dev.) of the measured values.

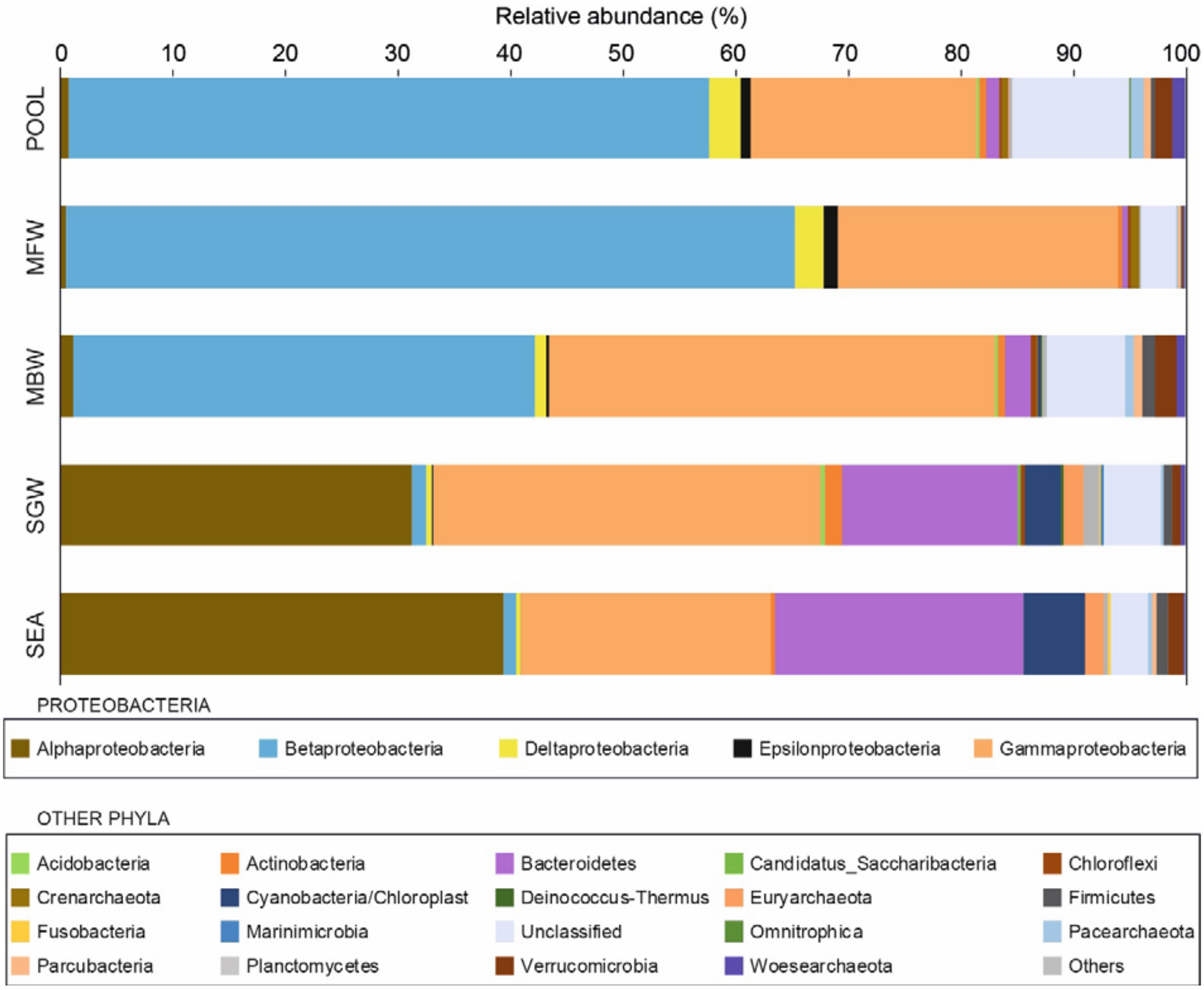

Supplementary Figure 4: Phylogenetic composition of the microbial community showing the relative abundance of phyla at the five water regimes. Proteobacteria is divided into classes Alpha-, Beta-, Gamma-, Delta- and Epsilonproteobacteria. The graph includes taxa that are grouped under 'Others' on **Fig. 4a** in main text.

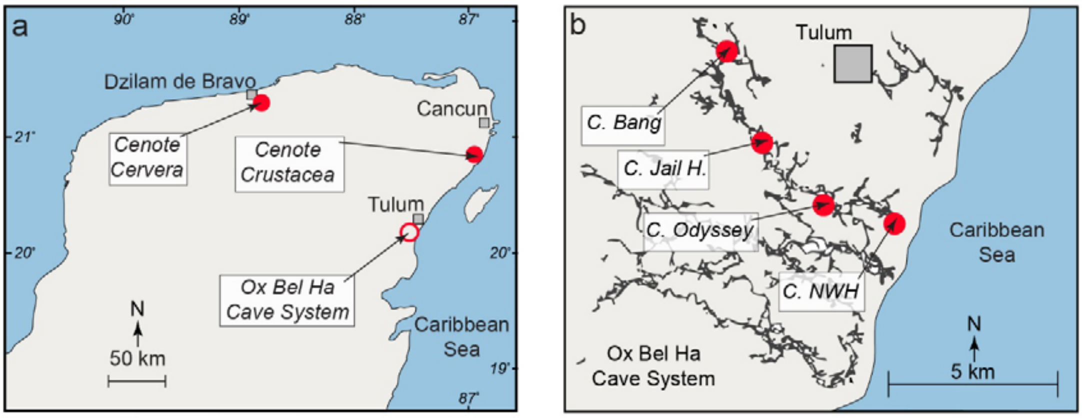

Supplementary Figure 5: Map of the **(a)** Yucatan Peninsula and the **(b)** Ox Bel Ha Cave System showing the sampling locations where *Typhlatya* spp. were collected for this study.

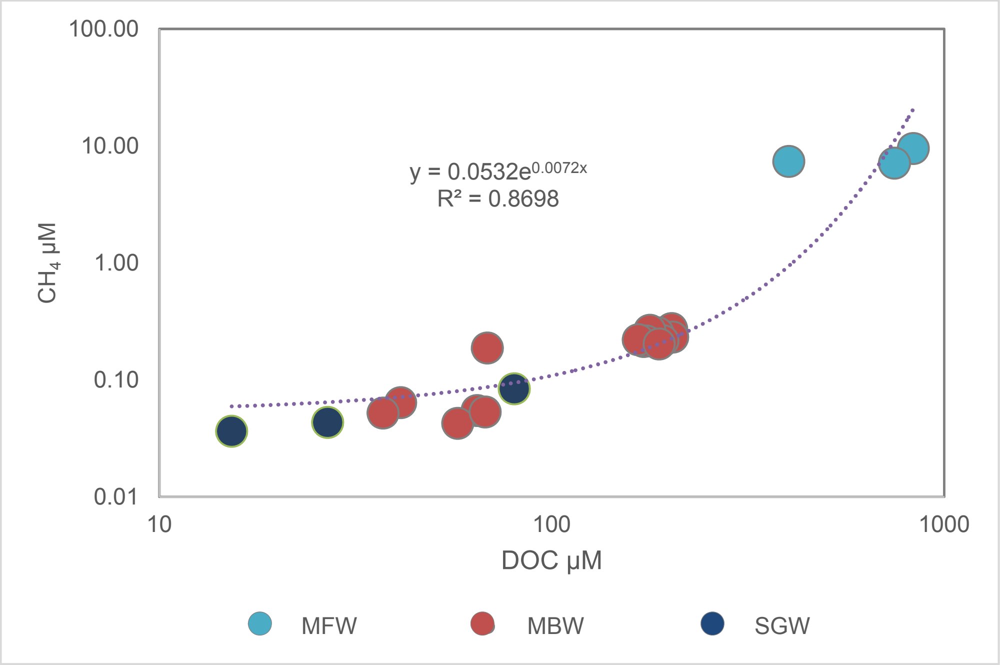

Supplementary Figure 6: Exponential relationship (dotted line) between methane and DOC concentrations in the water column of the flooded cave passages. Different colors refer to the water regimes (meteoric freshwater = MFW, meteoric brackish water = MBW, and saline groundwater = SGW).

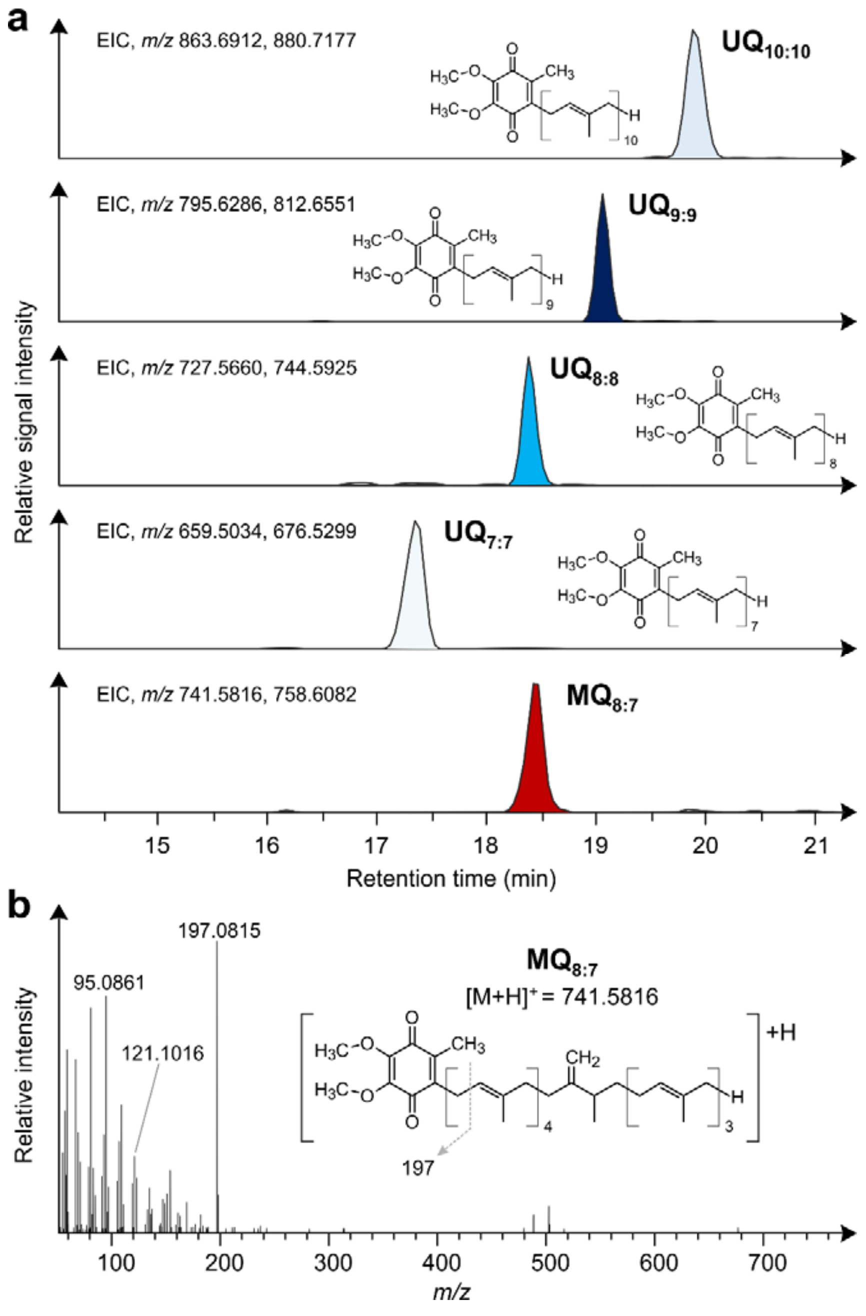

Supplementary Figure 7: Identification of quinones in sample 'P07 LIP' (see **Supplementary Data 1** for details). **(a)** Extracted ion chromatograms (EICs) of all identified quinones including molecular structures of ubiquinones (UQ). **(b)** Product ion (MS^2) spectra for methylene-ubiquinone ($MQ_{8:7}$, $[M+H]^+$ ion at *m/z* 741.6). The molecular structure and the formation of the major product ion are also shown. Quinone nomenclature ($Q_{m:n}$) is used, where Q indicates type of quinone, m the number of isoprenoid units in the side chain and n the number of double bonds.

sampling event	YSI sonde deployment	total precipitation (mm) 15 days prior	total precipitation (mm) 30 days prior
**2013 December**	December 5, 2013	457	906
**2014 August**	August 16, 2014	52	194
**2015 January**	January 13, 2015	39	133
**2016 January**	January 23, 2016	253	443

Supplementary Table 1: Total precipitation prior to YSI sonde deployment at the four sampling events based on data obtained from the closest available weather station (Cozumel International Airport). Weather database retrieved from https://www.wunderground.com/history/airport/MMCZ on January 7th, 2017.

**2013, December**					
MFW	MBW	SGW	POOL	SEA	
Salinity psu	0.25 (2)	1.98 ± 0.03 (9)	33.00 (1)	-	-
$[SO_4^{2-}]$ mM	0.20 (2)	1.59 ± 0.01 (9)	26.84 (1)	-	-
$[CH_4]$ nM	8323 (2)	233 ± 9 (9)	84 (1)	-	-
$\delta^{13}C$-CH_4 ‰	− 66.6 (2)	− 40.7 ± 0.5 (8)	− 51.2 (1)	-	-
[DOC] μM	791 (2)	185 ± 4 (9)	80 (1)	-	-
$\delta^{13}C$-DOC ‰	− 27.9 (2)	− 27.8 ± 0.1 (9)	− 27.2 (1)	-	-
[DIC] mM	4.5 (2)	6.9 ± 0.1 (9)	2.2 (1)	-	-
$\delta^{13}C$-DIC ‰	− 18.6 (2)	− 12.7 ± 1.7 (9)	− 0.1 (1)	-	-
**2014, August**					
MFW	MBW	SGW	POOL	SEA	
Salinity psu	0.26 (2)	1.52 ± 0.02 (7)	34.73 (2)	-	-
$[SO_4^{2-}]$ mM	0.52 (1)	1.40 ± 0.02 (7)	28.53 (2)	-	-
$[CH_4]$ nM	7513 (2)	76 ± 23 (6)	40 (2)	-	-
$\delta^{13}C$-CH_4 ‰	− 65.4 (1)	− 55.5 ± 1.5 (6)	− 52.9 (2)	-	-
[DOC] μM	402 (1)	62 ± 7 (7)	21 (2)	-	-
$\delta^{13}C$-DOC‰	− 28.2 (1)	− 29.0 ± 0.5 (7)	− 26.4 (2)	-	-
[DIC] mM	4.8 (1)	7.2 ± 0.6 (6)	2.2 (1)	-	-
$\delta^{13}C$-DIC ‰	− 15.6 (2)	− 8.8 ± 0.6 (7)	− 2.3 (2)	-	-
**2015, January**					
	MFW	MBW	SGW	POOL	SEA
Salinity psu	0.24 ± 0.03 (3)	1.88 ± 0.02 (9)	31.99 ± 1.46 (8)	0.73 ± 0.02 (3)	35.12 ± 0.78 (3)
$[SO_4^{2-}]$ mM	0.18 ± 0.02 (3)	1.68 ± 0.04 (9)	25.84 ± 1.26 (8)	0.59 ± 0.03 (3)	28.13 ± 0.68 (3)
$[CH_4]$ nM	5501 ± 411 (3)	137 ± 23 (9)	108 ± 16 (7)	168 ± 47 (3)	157 ± 44 (3)
$\delta^{13}C$-CH_4 ‰	− 65.4 ± 1.2 (3)	− 59.2 ± 1.3 (8)	− 56.1 ± 1.8 (6)	− 40.1 ± 3.3 (3)	− 60.8 ± 1.9 (3)
[DOC] μM	-	-	-	-	-
$\delta^{13}C$-DOC‰	-	-	-	-	-
[DIC] mM	4.2 ± 0.1 (3)	7.2 ± 0.2 (9)	2.4 ± 0.3 (8)	5.3 ± 0.2 (3)	2.0 ± 0.1 (3)
$\delta^{13}C$-DIC ‰	− 15.5 ± 0.2 (3)	− 11.2 ± 0.7 (9)	− 8.0 ± 0.4 (8)	− 9.4 ± 2.1 (3)	− 4.3 (2)
**2016, January**					
MFW	MBW	SGW	POOL	SEA	
Salinity psu	0.40 (1)	1.76 ± 0.03 (4)	34.49 (2)	1.14 ± 0.02 (3)	35.78 ± 0.21 (3)
$[SO_4^{2-}]$ mM	0.33 (1)	1.59 (2)	-	1.02 ± 0.01 (3)	29.28 (1)
$[CH_4]$ nM	3551 (1)	158 ± 63 (4)	203 (2)	821 ± 35 (3)	85 ± 25 (3)
$\delta^{13}C$-CH_4 ‰	− 69.2 (1)	− 62.0 ± 4.2 (3)	− 63.0 (2)	− 61.0 ± 0.2 (3)	− 56.2 (2)
[DOC] μM	-	-	-	-	-
$\delta^{13}C$-DOC‰	-		-	-	-
[DIC] mM	-	-	-	-	-
$\delta^{13}C$-DIC ‰	-	-	-	-	-

Supplementary Table 2: Summary of dissolved constituents for each sampling event. Values of constituents, presented as average ± std. error (n), from the different regimes of the groundwater system and the adjacent coastal sea. Water masses are MFW = meteoric freshwater; MBW = meteoric brackish water; SGW = saline groundwater; POOL = sinkhole pool; SEA = coastal sea water.

2015, June					
	MFW	MBW	SGW	POOL	SEA
POC µM	3.3 (1)	0.7 (1)	1.2 (1)	19.0 (2)	5.8 (1)
δ^{13}C-POC‰	− 29.4 (1)	− 26.21 (1)	− 25.04 (1)	− 28.28 (2)	− 20.12 (1)

2016, January					
	MFW	MBW	SGW	POOL	SEA
POC µM	14.6 (2)	7.2 (2)	4.0 (2)	58.7 (1)	-
δ^{13}C-POC‰	− 28.0 (2)	− 28.3 (2)	− 28.2 (2)	− 27.4 (1)	-

Supplementary Table 3: Summary of particulate organic carbon (POC) sampling events. Values, presented as measured value (n) or average (n), from the different regimes of the groundwater system and the adjacent coastal sea.

Bulk $\delta^{13}C$ of shrimp specimen = − 29.3 ‰		
**FA compound**	**Relative abundance**	**$\delta^{13}C$ of FA compound**
**$C_{14:0}$**	3.6%	− 31.56 ‰
**$10MeC_{14:0}$**	-	-
**$iC_{15:0}$**	10.0%	− 29.07 ‰
**$aiC_{15:0}$**	4.0%	− 29.29 ‰
**$C_{15:0}$**	2.0%	− 29.57 ‰
**$14Me\text{-}C_{15:0}$**	4.2%	− 29.58 ‰
**$C_{16:1\omega9}$**	1.9%	− 29.95 ‰
**$C_{16:1\omega7c}$**	7.5%	− 36.84 ‰
**$C_{16:1\omega5c\&t}$**	4.4%	− 32.26 ‰
**$C_{16:0}$**	23.6%	− 30.49 ‰
**$10Me\text{-}C_{16:0}$**	3.2%	− 29.16 ‰
**$9MeC_{16:0}$**	-	-
**$8MeC_{16:0}$**	-	-
**$iC_{17:0}$**	3.5%	− 29.71 ‰
**$_{ai}C17:0$**	3.1%	− 29.02 ‰
**$cyC_{17:0\omega5,6}$**	1.3%	− 32.05 ‰
**$C_{17:0}$**	1.7%	− 30.30 ‰
**$C_{18:2}$**	1.3%	− 33.17 ‰
**$C_{18:1\omega9}$**	10.2%	− 29.68 ‰
**$C_{18:1\omega7c}$**	6.9%	− 32.48 ‰
**$C_{18:1\omega5}$**	0.9%	− 29.30 ‰
**$C_{18:0}$**	6.9%	− 29.13 ‰

Bulk $\delta^{13}C$ of shrimp specimen = − 49.1 ‰		
**FA compound**	**Relative abundance**	**$\delta^{13}C$ of FA compound**
$C_{14:0}$	3.0%	− 54.69 ‰
$10MeC_{14:0}$	3.0%	− 67.01 ‰
$iC_{15:0}$	1.0%	− 42.00 ‰
$aiC_{15:0}$	1.2%	− 45.56 ‰
$C_{15:0}$	0.9%	− 47.06 ‰
$14Me\text{-}C_{15:0}$	0.9%	− 49.03 ‰
$C_{16:1\omega9}$	-	-
$C_{16:1\omega7c}$	20.0%	− 54.12 ‰
$C_{16:1\omega5c\&t}$	1.7%	− 51.58 ‰
$C_{16:0}$	28.8%	− 51.58 ‰
$10Me\text{-}C_{16:0}$	2.2%	− 55.06 ‰
$9MeC_{16:0}$	0.7%	− 54.78 ‰
$8MeC_{16:0}$	0.6%	− 58.35 ‰
$iC_{17:0}$	0.8%	− 52.70 ‰
$_{ai}C17:0$	1.3%	− 44.67 ‰
$cyC_{17:0\omega5,6}$	0.8%	− 49.49 ‰
$C_{17:0}$	0.8%	− 52.79 ‰
$C_{18:2}$	-	-
$C_{18:1\omega9}$	17.6%	− 52.48 ‰
$C_{18:1\omega7c}$	8.3%	− 50.78 ‰
$C_{18:1\omega5}$	0.7%	− 48.22 ‰
$C_{18:0}$	5.6%	− 48.60 ‰

Supplementary Table 4: Full suite of fatty acid (FA) compounds extracted from the tissue of two *Typhlatya* shrimp specimens shown on **Fig. 5b** in main text.

sample ID	sampling site	δD ‰ ± std.dev.		$\delta^{13}C$ ‰ ± std.dev.		water mass	% CH_4-derived carbon
**CB-02**	Cenote Bang	-		− 29.27	± 0.14	MBW	3%
**CB-04**	Cenote Bang	-		− 49.13	± 0.11	MBW	55%
**CB-05**	Cenote Bang	− 152.35	± 1.60	− 38.56	± 0.05	MBW	28%
**CB-06**	Cenote Bang	− 134.01	± 1.60	− 36.68	± 0.05	MBW	23%
**CB-07**	Cenote Bang	− 124.97	± 1.60	− 31.82	± 0.05	MBW	10%
**CB-08**	Cenote Bang	− 192.95	± 1.60	− 43.65	± 0.05	MBW	41%
**CB-09**	Cenote Bang	− 141.03	± 1.60	− 32.80	± 0.05	MBW	13%
**CB-10**	Cenote Bang	− 150.49	± 1.60	− 31.74	± 0.05	MBW	10%
**CB-11**	Cenote Bang	− 95.73	± 1.60	− 34.99	± 0.05	MBW	18%
**CB-12**	Cenote Bang	− 146.68	± 1.60	− 35.01	± 0.05	MBW	18%
**CB-13**	Cenote Bang	− 114.44	± 1.60	− 29.35	± 0.05	MBW	4%
**CB-14**	Cenote Bang	− 98.75	± 1.60	− 30.52	± 0.05	MBW	7%
**JH-02**	Cenote Jail House	− 147.67	± 1.90	− 26.94	± 0.36	MBW	0 %
**JH-03**	Cenote Jail House	− 119.51	± 1.90	− 33.32	± 0.36	MBW	14%
**CO-02**	Cenote Odyssey	− 180.38	± 1.90	− 22.52	± 0.36	SGW	0 %
**TA-01**	Cenote N.W.H.	− 195.02	± 1.90	− 33.33	± 0.36	MBW	14%
**TA-02**	Cenote N.W.H.	− 158.11	± 1.90	− 34.28	± 0.36	MBW	16%
**TA-04**	Cenote N.W.H.	− 197.17	± 1.90	− 44.99	± 0.36	MBW	44%
**CC-06**	Cenote Crustacea	− 150.08	± 1.90	− 32.78	± 0.36	MBW	12%
**CC-07**	Cenote Crustacea	− 219.22	± 1.90	− 41.39	± 0.36	SGW	35%
**CC-08**	Cenote Crustacea	− 171.24	± 1.90	− 41.39	± 0.36	MBW	35%
**CC-09**	Cenote Crustacea	− 215.93	± 1.90	− 45.30	± 0.36	SGW	45%
**CC-10**	Cenote Crustacea	− 223.64	± 1.90	− 42.29	± 0.36	SGW	37%
**CC-11**	Cenote Crustacea	− 185.37	± 1.90	− 38.45	± 0.36	MBW	27%
**CC-12**	Cenote Crustacea	− 164.71	± 1.90	− 32.92	± 0.36	SGW	13%
**CC-16**	Cenote Crustacea	− 205.29	± 1.90	− 37.51	± 0.36	MBW	25%
**DZ-01**	Cenote Cervera	− 189.12	± 1.90	− 42.96	± 0.10	SGW	39%
**Mean value ± std. error:**		**− 162.96**	**± 7.38**	**− 36.07**	**± 1.21**	**-**	**21%**

Supplementary Table 5: Collected specimens (*Typhlatya* spp.), listed with the sampling site and type of water mass where the specimens were found, as well as their bulk δD and $\delta^{13}C$ values. Calculated percent CH_4-derived carbon in the biomass of the individual shrimp is also reported.

PROCESSING WATER SAMPLES COLLECTED WITH 60 ML SYRINGES

**subsample analysis**	CH_4 conc. & $\delta^{13}C$-CH_4	DIC conc.	$\delta^{13}C$-DIC	ions	DOC conc. & $\delta^{13}C$-DOC
**order processed in the field lab**	1	2	3	4	5
**vial volume**	30 ml	5 ml	2 ml	1.5 ml	8 ml
**vial type**	glass serum	glass serum	glass serum	plastic	glass
**vial cleaning**	baked 450 °C / 4 hrs	baked 450 °C / 4 hrs	baked 450 °C / 4 hrs	milli-Q rinse	baked 450 °C / 4 hrs
**septa / cap**	butyl rubber	Teflon	Teflon	plastic	Teflon
**septa / cap cleaning**	milli-Q rinse	-	-	milli-Q rinse	acid wash
**subsample volume**	~ 20 ml	3 ml	> 1.5 ml	> 1 ml	> 6 ml
**filter used***	no	no	yes	yes	yes
**field storage**	fridge	freezer	freezer	fridge	freezer
**transportation**	cool	blue ice	blue ice	cool	dry ice
**storage temp.**	< 7 °C	− 20 °C	− 20 °C	< 7 °C	− 20 °C
**analytical method**	GC & GC-IRMS	Coulometer	GC-IRMS	IC	HTC-IRMS

Supplementary Table 6: Water samples collected manually with 60 ml syringes were processed in the field within 8 hours after collection. Processing included dividing each sample into subsamples in the order shown above (left to right). *Filter used was a 0.45 µm Acrodisc® inorganic membrane syringe filter.
