# Supplementary material for: Methane- and dissolved organic carbon-fueled microbial loop supports a tropical subterranean estuary ecosystem
Source: Nat Commun. 2017 Nov 28;8:1835. doi: 10.1038/s41467-017-01776-x (PMC5703975; doi:10.1038/s41467-017-01776-x)
Supplement: Supplementary file 3 — Description of Additional Supplementary Files [file 41467_2017_1776_MOESM3_ESM.pdf]

Description of Additional Supplementary Files

File Name: Supplementary Data 1
Description: Details of the samples used in the study.

File Name: Supplementary Data 2
Description: All observed phylogenetic affiliations based on sequence reads from 16S RNA genes.
